# Cognitive dysfunctions and psychological symptoms in migraine without aura: a cross-sectional study

**DOI:** 10.1186/s10194-016-0667-0

**Published:** 2016-08-27

**Authors:** Gabriella Santangelo, Antonio Russo, Luigi Trojano, Fabrizia Falco, Laura Marcuccio, Mattia Siciliano, Francesca Conte, Federica Garramone, Alessandro Tessitore, Gioacchino Tedeschi

**Affiliations:** 1 Department of Psychology, Second University of Naples, Caserta, 81100 Italy; 2 Headache Center, Department of Medical, Surgical, Neurological, Metabolic and Aging Sciences, Second University of Naples, Naples, 80138 Italy; 3 Institute for Diagnosis and Care “Hermitage Capodimonte”, Naples, 80100 Italy; 4 Salvatore Maugeri Foundation, Scientific Institute of Telese, Telese Terme, BN 82037 Italy

**Keywords:** Migraine, Cognitive deficits, Depression, Apathy, MoCA, Montreal cognitive assessment

## Abstract

**Background:**

The occurrence of cognitive dysfunctions and psychological symptoms, as well as their mutual relationships, in migraine patients are still debated. The aim of the study was to characterize the cognitive profile and psychological symptoms (i.e. depression, anxiety and apathy) in drug-naïve migraine without aura (MwoA) patients.

**Methods:**

Seventy-two consecutive MwoA patients, referred to the Italian University Headache Clinic and 72 healthy subjects (HCs) were enrolled. Patients, during an attack-free period, and HCs completed Montreal Cognitive Assessment (MoCA), Beck Depression Inventory-II (BDI-II), Self-version of Apathy Evaluation Scale (AES-S) and State and Trait Anxiety Inventory (STAI-Y-1 and 2). Clinical parameters of disease severity (i.e. disease duration, migraine attacks per month, mean pain intensity during migraine attacks, migraine disability and impact on daily life) were recorded.

**Results:**

Although performance of MwoA patients on MoCA was above Italian cut-off threshold (<15.5) suggesting presence of cognitive impairment, MwoA patients achieved significantly lower scores than HCs on total MoCA scale (22.3 ± 2.7 versus 25.4 ± 2.3) and on its attention (4.9 ± 1.1 versus 5.6 ± 0.7), memory (1.8 ± 1.4 versus 3.1 ± 1.3), visuospatial (3.2 ± 0.9 versus 3.6 ± 0.6) and executive subscales (2.6 ± 1.1 versus 3.1 ± 0.8). In addition, we observed significant correlations between MoCA executive domain subscore and the attack-related disability score (MIDAS).

As for behavioral profile, the percentage of depressive symptoms (4.2 %), high state and trait anxiety (13.9 and 9.7 %, respectively), and apathy (11.1 %) in MwoA patients were similar to that of HCs. No significant associations of behavioural symptoms with cognitive performance and clinical parameters were found.

**Conclusions:**

Drug-naïve MwoA patients are characterized by subtle cognitive dysfunctions and low percentage of behavioural symptoms. The results support the importance of searching for subclinical cognitive disturbances in patients with MwoA, who deserve to be followed-up to verify whether they develop clinically relevant disorders over time.

## Background

Migraine is one of the most common pain disorders with a prevalence of 5-20 % in the general population [1], higher in women than in men (with an average prevalence of 20.2 % versus 9.4 %) [2]. Until now, the relationship between migraine and cognitive deficits has been investigated by several cross-sectional studies providing divergent results (for a review see 3). Indeed, some studies did not find any cognitive difference between migraine patients and non-migraine subjects [4–7]. Conversely, other studies revealed that migraine patients are characterized by a poorer cognitive performance [8–15] during both interictal [11–13] or ictal [16, 17] phases when compared to HCs. In particular, it has been suggested that migraine patients might show selective defects in executive/attention and visuospatial domains [9, 11, 13–15].

The discrepancies among studies assessing cognitive functions in migraine may be ascribed to several reasons, possibly related to differences in patients’ characteristics (some studies enrolled both patients with or without aura), sample sizes or neuropsychological assessments [3]. Moreover, some studies did not control the possible influence of clinical variables, such as frequency of attacks, or relationship with pharmacological treatment. In fact, no neuropsychological study specified the role of pharmacological treatment even though some treatments for migraine (e.g., topiramate, amytriptiline) have been associated with cognitive dysfunctions [18, 19]. Last, only a few studies took into account the possible relationships between cognitive performances and associated psychological symptoms or behavioural disturbances, which may often occur in migraine patients [20]. Migraine patients may also present affective temperamental dysregulation, with high hopelessness that may be considered a significant risk factor for negative outcome [21]. Among behavioural disturbances, apathy appears not only as a symptom of depression but as a specific behavioural disturbance [22], with its neural (i.e. abnormalities of prefrontal cortex) and cognitive correlates (i.e. impaired executive/attention and visuospatial abilities) [23, 24]. Since previous neuroimaging studies revealed reduced functional connectivity in the fronto-parietal network in MwoA patients [25], and apathy is associated mainly with abnormalities of prefrontal cortex, it is possible that apathy in itself, not associated with clinically relevant depression, can occur in MwoA. Nonetheless, until now no study specifically assessed apathy, i.e. a loss of interest and motivation [22], in MwoA.

On the basis of the above considerations, the present study aimed at investigating the cognitive profile in a homogeneous sample of drug-naïve migraine without aura (MwoA) patients, using the Montreal Cognitive Assessment (MoCA) [26], a widely available screening tool, easy to use in clinical practice. Due to its sensitivity to executive/attention and visuospatial impairments, MoCA seems particularly suitable as a neuropsychological assessment tool in migraine patients, who might show selective defects in such cognitive domains [9, 11, 13–15]. Moreover, we investigated possible relationships between cognitive performances and psychological symptoms such as depression, anxiety and apathy. Finally, we compared neuropsychological and psychological pattern in these patients with those of a group of healthy controls (HCs) matched for age and educational level to patients.

## Methods

### Subjects

In the present study, consecutive patients with diagnosis of MwoA were recruited from migraine population referring to the outpatient Headache Clinic of the First Division of Neurology of the University of Naples, Italy, from September 2014 to June 2015. The inclusion criterion to participate in the present study was a diagnosis of MwoA according to the ICHD-III beta version criteria of the International Headache Society (IHS). Moreover, we did not enrol in the study patients who showed one of the following exclusion criteria: 1) other ICHD-III diagnosis (e.g., tension type headache, chronic migraine etc.), somatic or psychiatric disorders (e.g., major depression, or psychosis according to DSM-V criteria); 2) current or previous intake of any pharmacological anti-migraine preventive therapy [27] (i.e., we selected patients drug-naïve for anti-migraine preventive treatment); 3) symptoms compatible with acute confusional migraine during migraine attacks. Moreover, to avoid any possible interference related to migraine attacks or to pharmacological treatment on cognitive functions, all MwoA patients were migraine free, and not taking rescue medications, for at least 3 days before and after the neuropsychological assessment. For this aim, patients were interviewed 3 days after neuropsychological assessment to ascertain this point.

After enrolment of MwoA patients, for each patient we selected a healthy individual with the same demographic features. MwoA patients and HCs were matched on age and education but not for gender, although the difference in gender distribution in the two samples was not statistically significant (Chi-squared 0.298; *p* = 0.585). HCs were recruited from among patients’ friends, employees at the clinic or university centres, and were included if they gave their written informed consent to participate on a voluntary basis and met the following selection criteria: lack of history of migraine or any other type of headache and/or current diagnosis of migraine according to clinical criteria; lack of history of or actual psychiatric diseases (e.g., major depression, or psychosis according to DSM-V criteria); no use of psychoactive drugs.

All selected participants gave their written informed consent to participate to the study, which was approved by the Local Ethic Committee and was performed in accordance with the ethical standards laid down in the 1964 Declaration of Helsinki.

### Procedures

The demographic and clinical aspects such as disease duration, migraine attacks per month, mean pain intensity during migraine attacks (by means of visual analogic scale -VAS) were recorded. Moreover, to obtain an accurate assessment of patients’ headache-related disability, MwoA patients completed the Migraine Disability Assessment Scale (MIDAS) [28] and the Headache Impact Test −6 (HIT-6) [29].

All patients and HCs completed Beck Depression Inventory-II (BDI-II) [30], Self-version of Apathy Evaluation Scale (AES-S) [22] and State and Trait Anxiety Inventory (STAI-Y-1 and STAI-Y-2, respectively) [31, 32].

The BDI-II is a questionnaire consisting of 21 items, designed to assess severity of depressive symptoms (e.g., sense of failure, guilt, social withdrawal, insomnia, or weight loss). The total score ranges from 0–63, with higher scores reflecting higher levels of depression. The questionnaire allows identifying patients with clinically significant depression according to well-known cut-off in general population (14–19 for mild depression, 20–28 for moderate depression and 29–63 for severe depression) [30].

The AES-S was developed to assess severity of apathy. The questionnaire includes 18 items evaluating four aspects of apathy: “behavioural”, “emotional”, “cognitive” and “other”. Items labeled “other” evaluate reduction or loss of motivation, initiative and accurate understanding of one’s problems. The total score ranges from 18 to 72. Clinically significant apathy was identified according to cut-off score of 38 [22].

The STAI-Y consists of two separate self-report scales for measuring state anxiety, intended as a transitory emotional state, and trait anxiety, consisting in stable tendency to attend to negative emotions such as fears or and anxiety across many situations. The total scores of both subscales (STAI-Y-1 and STAI-Y-2, respectively) range 20–80, and are converted to T-score; a T-score higher than 65 on STAI-Y-1 and STAI-Y-2 indicated high level of state and trait anxiety [31, 32].

All patients and HCs underwent the Italian version of Montreal Cognitive Assessment (MoCA) [26] to evaluate global cognitive status and several cognitive domains: memory, attention, language, and orientation, visuospatial and executive functions domains. The MoCA total score ranges from 0 (worst performance) to 30 (best performance). According to the Italian norms a value of age- and education-adjusted total MoCA score lower than 15.5 is suggestive for presence of cognitive decline; the Italian norms also provide cut-off values for five cognitive domains [33].

### Statistical analysis

The comparison between MwoA patients and HCs on demographic, neuropsychiatric and cognitive aspects was performed by means of multivariate analysis of variance (MANOVA) and Chi-squared, as appropriate. The performance of MwoA patients and HCs on MoCA was also compared to Italian normative data for MoCA and single cognitive domains to identify how many individuals had clinically relevant cognitive impairment [33].

Several procedures (i.e. Shapiro-Wilk normality test, kurtosis and skewness and a z-score obtained by dividing the skew values or excess kurtosis by their standard errors) to evaluate normal distribution of cognitive and behavioural variables. Within the sample of MwoA patients, the association between clinical, neuropsychiatric and cognitive variables was carried out by means of Spearman’s rank correlation coefficient. Although value of *p* < 0.05 was considered statistically significant, the Bonferroni correction for multiple comparisons was applied.

All analyses were performed using SPSS version 20, (SPSS Inc., Chicago, IL, USA).

## Results

Eighty consecutive patients with diagnosis of MwoA were screened, but only 72 MwoA patients (63 females and 9 males) were enrolled, since 8 patients experienced migraine attacks during the 3 days following the neuropsychological assessment. Moreover, 72 HCs were included in the present study (see Table 1 for a summary of demographic and clinical features).

**Table 1:** Demographic and clinical aspects of migraineurs without aura (MwoA) and healthy subjects

	MwoA	Healthy subjects	*χ* ^2^/F	*P*
Gender (Females/Males)	63/9	66/6	0.298	0.585
Age (years)	34.9 ± 11.2	33.8 ± 11.9	0.421	0.193
Education (years)	12.1 ± 3.6	11.9 ± 3.8	0.112	0.738
Disease duration (years)	15.1 ± 11.6 (13.5; 16.7)	-	-	-
Attacks per month	6.1 ± 5.1 (4; 5)	-	-	-
MIDAS score	25.3 ± 19.5 (20; 25.75)	-	-	-
HIT-6 score	59.9 ± 8.7 (60.5; 10)	-	-	-
VAS score	11.1 ± 12.5 (8.2; 10.7)	-	-	-
Side of pain				
Left	10	-		
Right	6	-		
Bilateral	56	-		

The values are expressed in Mean ± Standard Deviation, median and interquartile range are reported in brackets; *MIDAS* Migraine Disability Assessment Scale, *HIT*-6 the Headache Impact Test −6, *VAS* Visual Analogic Scale

### Neuropsychological assessment

MANOVA showed that MwoA patients and HCs had different cognitive profiles (Wilks’ Lambda = 0.694, F = 8.559, df = 7, *p* < 0.001). MwoA patients performed significantly lower than HCs on the total MoCA score, and on attention, memory, visuospatial and executive domains (all *p* < 0.007 after Bonferroni correction). No significant difference between the two groups was found on language and orientation domains (Table 2).

**Table 2:** Cognitive comparison between migraineurs without aura (MwoA) and healthy subjects

	MwoA	Healthy subjects	F	*P*
	Mean ± SD	Mean ± SD		
MoCA adjusted total score	22.3 ± 2.7	25.4 ± 2.3	56.606	**<0.001**
Cognitive Domains				
Visuospatial functions	3.2 ± 0.9	3.6 ± 0.6	12.865	**<0.001**
Executive functions	2.6 ± 1.1	3.1 ± 0.8	11.297	**0.001**
Attention	4.9 ± 1.1	5.6 ± 0.7	14.776	**<0.001**
Language	5.2 ± 0.8	5.6 ± 0.6	7.452	0.007
Memory	1.8 ± 1.4	3.1 ± 1.3	29.660	**<0.001**
Orientation	5.9 ± 0.2	6 ± 0	5.299	0.023

*SD* Standard Deviation, *MoCA* Montreal Cognitive Assessment

In bold are reported significant differences after Bonferroni correction

With reference to available normative data for MOCA, no MwoA individual and no control achieved a total score below normal range.

### Behavioural assessment

MANOVA did not reveal significant difference between MwoA patients and HCs on the behavioural profile (Wilks’ Lambda = 0.940; F = 1.077, df =8; *p* = 0.383), as assessed by BDI-II, AES-S and its subscales, STAY-1 and STAY-2 (Table 3).

**Table 3:** Behavioural comparisons between migraineurs without aura (MwoA) and healthy subjects

	MwoA (Mean ± SD)	Healthy Subjects (Mean ± SD)	F	*P*
BDI-II	10.6 ± 9.4	8.9 ± 6.4	1.708	0.193
AES-S: behavioral subscale	7.8 ± 2.5	7.9 ± 2	0.033	0.856
AES-S: cognitive subscale	12.6 ± 3.2	13.5 ± 2.9	2.624	0.107
AES-S: emotive subscale	3.6 ± 1	3.6 ± 1.2	0.022	0.883
AES-S: “others” subscale	6.1 ± 1.6	6.1 ± 1.4	0	1.000
AES-S: total score	29.9 ± 5.5	30.5 ± 5.4	0.351	0.555
STAI-Y-1	40.6 ± 11.6	39.2 ± 12.1	0.453	0.502
STAI-Y-2	42.1 ± 9.7	39.6 ± 9.7	2.324	0.130
Severity of Depressive symptoms (cut-off score of BDI-II)				
Severe	3 (4.2 %)	1 (1.7 %)		
Moderate	7 (9.7 %)	2 (2.7 %)		
Mild	8 (11.1 %)	10 (13.8 %)		
No depression	54 (75 %)	59 (81.8 %)		
Levels of state anxiety (cut-off score of STAY-I-1)				
High	10 (13.9 %)	8 (11.1 %)		
Levels of trait anxiety (cut-off score of STAY-I-2)				
High	7 (9.7 %)	3 (4.2 %)		
Levels of apathy (cut-off score of AES-S)				
High	8 (11.1 %)	7 (9.7 %)		

*SD* Standard Deviation, *BDI-II* Beck Depression Inventory-II, *AES-S* Self-version of Apathy Evaluation Scale, *STAI-Y* State-Trait Anxiety Inventory- Y

With reference to available cut-off scores, severity of depressive symptoms (*χ*^2^ = 4.221; df = 2; *p* = 0.239), of levels of state and trait anxiety (both *χ*^2^ < 1) and of apathy (*χ*^2^ < 1) did not differ in MwoA and HC groups (Table 3).

### Correlation analysis between clinical, cognitive and neuropsychiatric variables in MwoA

We did not observe significant relationship between total MoCA score or subdomain scores with behavioural scores (AES-S, BDI-II and STAI-Y-1 and STAI-Y-2) (Table 4).

**Table 4:** Correlation between cognitive scores and behavioural scores in patients with migraine without aura

	BDI-II	AES-S -Behaviour	AES-S-Cognitive	AES-S-Emotive	AES-S-Others	AES-S-total	STAI-Y-1	STAI-Y-2
Cognitive domains	r (*p* value)	r (*p* value)	r (*p* value)	r (*p* value)	r (*p* value)	r (*p* value)	r (*p* value)	r (*p* value)
Visuospatial	−0.0162 (0.174)	−0.062 (0.603)	0.012 (0.923)	−0.133 (0.267)	0.017 (0.887)	−0.028 (0.815)	−0.012 (0.918)	−0.071 (0.554)
Executive	−0.180 (0.130)	−0.043 (0.722)	−0.042 (0.727)	0.047 (0.697)	0.099 (0.409)	0.005 (0.965)	−0.178 (0.134)	−0.129 (0.282)
Attention	0.016 (0.894)	−0.096 (0.421)	0.009 (0.939)	0.134 (0.262)	0.099 (0.408)	0.045 (0.707)	0.048 (0.688)	0.081 (0.499)
Language	−0.088 (0.462)	0.063 (0.601)	−0.220 (0.063)	0.032 (0.789)	0.283 (0.016)	−0.040 (0.737)	−0.026 (0.826)	0.049 (0.680)
Memory	−0.162 (0.175)	−0.149 (0.213)	−0.153 (0.199)	0.104 (0.386)	0.119 (0.320)	−0.070 (0.560)	−0.123 (0.304)	−0.067 (0.574)
Orientation	0.166 (0.164)	0.129 (0.280)	0.239 (0.043)	0.265 (0.024)	0.139 (0.245)	0.284 (0.015)	0.024 (0.840)	0.109 (0.361)
MoCA total score	−0.75 (0.533)	−0.033 (0.785)	0.102 (0.393)	0.218 (0.065)	0.181 (0.128)	−0.004 (0.972)	−0.130 (0.276)	−0.072 (0.583)

*BDI-II* Beck Depression Inventory-II, *AES-S* Apathy Evaluation Scale-Self version, *MoCA* Montreal Cognitive Assessment, *STAI-Y* State-Trait Anxiety Inventory- Y, r indicates Pearson's correlation coefficient

After Bonferroni correction, MoCA-executive domain score showed a significant, negative and moderate correlation with MIDAS score whereas the remaining cognitive measures did not correlate with clinical and neuropsychiatric variables (Table 5).

**Table 5:** Correlation between behavioral, cognitive and clinical parameters in patients with migraine without aura

	Disease duration	Attacks per month	MIDAS	HIT-6	VAS
Cognitive domains	rho (*p* value)	rho (*p* value)	rho (*p* value)	rho (*p* value)	rho (*p* value)
Visuospatial	−0.117 (0.342)	0.139 (0.252)	0.067 (0.589)	0.177 (0.149)	0.113 (0.359)
Executive	0.177 (0.149)	−0.307 (0.010)	**−0.341 (0.004)**	−0.092 (0.455)	0.098 (0.427)
Attention	0.099 (0.421)	−0.011 (0.931)	0.043 (0.729)	0.221 (0.070)	0.041 (0.742)
Language	0.061 (0.620)	−0.128 (0.290)	−0.248 (0.041)	−0.079 (0.524)	0.116 (0.348)
Memory	−0.205 (0.094)	−0.008 (0.950)	−0.112 (0.361)	−0.151 (0.218)	0.196 (0.109)
Orientation	0.197 (0.108)	0.111 (0.359)	0.088 (0.477)	0.187 (0.126)	0.055 (0.654)
MoCA total score	−0.021 (0.865)	−0.006 (0.963)	−0.093 (0.453)	0.160 (0.192)	0.317 (0.008)
BDI-II	0.134 (0.275)	0.031 (0.799)	0.254 (0.037)	0.141 (0.251)	−0.065 (0.601)
AES-S Behaviour	0.056 (0.648)	0.232 (0.053)	0.061 (0.623)	0.110 (0.372)	0.029 (0.817)
AES-S-Cognitive	0.007 (0.957)	0.187 (0.121)	0.210 (0.085)	0.298 (0.014)	−0.037 (0.763)
AES-S-Emotive	0.059 (0.632)	0.022 (0.855)	−0.011 (0.930)	−0.057 (0.645)	−0.147 (0.231)
AES-S-Others	−0.030 (0.809)	−0.078 (0.521)	−0.017 (0.894)	0.010 (0.936)	−0.261 (0.031)
AES-S-Total	0.059 (0.634)	0.197 (0.102)	0.180 (0.141)	0.207 (0.090)	−0.115 (0.352)
STAI-Y-1	0.041 (0.742)	0.015 (0.900)	0.076 (0.535)	−0.091 (0.462)	−0.200 (0.102)
STAI-Y-2	0.126 (0.307)	0.070 (0.563)	0.156 (0.205)	−0.060 (0.626)	−0.009 (0.944)

*MIDAS* Migraine Disability Assessment Scale, *HIT-6* the Headache Impact Test −6, *VAS* Visual Analogic Scale, *BDI-II* Beck Depression Inventory-II, *AES-S* Apathy Evaluation Scale-Self version, *MoCA* Montreal Cognitive Assessment, *STAI-Y* State-Trait Anxiety Inventory-Y, rho indicates Spearman rho correlation

Significant correlation was reported in bold after Bonferroni correction (for cognitive variables: 0.004 (0.05/12); for behavioural variable: 0.003 (0.05/13)

## Discussion

The present study revealed that MwoA patients had significantly lower scores than HCs on the total MoCA scale and in 4 out of 6 cognitive subdomains in all subscores (i.e., executive function, attention, visuospatial and memory domains). However, no MwoA patients achieved scores below the available cut-off values, thus suggesting that the reduced efficiency in selected cognitive domains did not correspond to a clinically relevant cognitive deterioration.

As for the psychological profile, MwoA patients and HCs reported similar scores for depression (BDI-II), trait and state anxiety (STAI-Y-1 and STAI-Y-2), and apathy (AES-S). Cognitive scores in MwoA patients were not found to be associated with severity of psychological disturbances, whereas depression, trait and state anxiety and apathy were associated among them.

Our first main finding demonstrated the occurrence of cognitive dysfunctions in MwoA patients who had never taken anti-migraine preventive drugs in the course of their life with respect to HCs. In particular, our results revealed lower scores in the subscales assessing verbal memory, attention, frontal and visuospatial functions, in line with several previous studies [8–15]. The low score on the memory scale (i.e., poor retrieval abilities) might be ascribed to defective strategic and organizational aspects of learning [9], also in consideration of the lower attentional and executive function scores in MwoA patients compared to HCs. However, previous studies evaluating migraine patients in the community did not find differences between MwoA patients and HCs [4–6]. The divergence between those studies and ours might be ascribed to the different patient selection procedures: in those studies MwoA patients were selected from a cohort of subjects (i.e., population-based register) on the basis of self-report measures. This procedure might have led to a misclassification of the non-migraine subjects, as suggested by Elkind and Scher [34], and to the enrolment of individuals with very mild migraine, in terms of frequency and intensity of attacks. Instead, we performed a study in a clinical setting where MwoA patients, identified by expert neurologists according to established clinical criteria, required medical intervention due to migraine and related disability. However, our results underlined that MwoA patients’ scores were not lower than the cut-off value reported in normative studies [25, 35]. We can, thus, suggest that MwoA patients with migraine symptoms needing medical consultations show “sub-clinical” neuropsychological impairments mainly affecting executive functions, i.e. a reduced efficiency of cognitive processes in the lack of clinically relevant cognitive deterioration.

Such cognitive results would be compatible with recent findings showing reduced functional connectivity in the fronto-parietal network even in migraineurs without overt executive dysfunctions, i.e. with scores on executive tests above the normal cut-off values [25]. It is also worth mentioning that in MwoA patients reduced connectivity in prefrontal and temporal regions of the default mode network [35], in the absence of structural abnormalities, thus suggesting that brain areas involved in executive control may show signs of dysfunction even before development of clinically detectable cognitive impairments.

In the present study we found no significant difference on severity of depression, apathy, and state and trait anxiety in MwoA patients compared to HCs. Prevalence of depression and anxiety was lower than that reported in previous studies in which depression, anxiety disorders and migraine were considered to be comorbid diseases [20]. Moreover, where previous study revealed a relationship between depression and migraine [36], we failed to find any association; the divergence among other studies and ours might depend on difference in inclusion and exclusion criterion and on employment of different tools to assess depression. However, our observation is in line with an epidemiological study supporting the idea that psychiatric comorbidity occurs less frequently in MwoA than in migraine with aura [37] or probable medication-overuse headache [38].

Clinically significant apathy occurred in 8 patients only (11 %), and we did not observe significant differences on apathy scale (AES-S) between patients and HCs. Although the finding of mild frontal/executive dysfunction in our patient sample might predict a higher frequency of apathy [23], it must be considered that we enrolled relatively young individuals and that apathy is thought to increase with age [39]. Since this is the first study assessing apathy in MwoA patients, we believe that this issue deserves further cross-sectional and longitudinal investigation in MwA and MwoA patients, who are both characterised by functional abnormalities in prefrontal cortex [25, 35].

Moreover, future studies in large samples of controls and migraineurs should investigate whether psychological disturbance modify the effect of migraine on cognitive impairment.

In the present study, several clinical aspects of migraine (pain intensity, disease duration, and frequency of attack) did not influence cognitive performance on MoCA. The lack of significant relationships between frequency of migraine attacks and cognitive performance is in agreement with most previous studies [10, 11], as is the lack of a significant correlation between disease duration and cognitive dysfunctions [9, 11], thus supporting the hypothesis that subtle alterations in information processing mechanisms might be present also in the early stages of migraine [11]. Finally, among clinical aspects of migraine, we found that high levels of attack related disability were associated with reduced executive functioning as assessed by the MoCA executive subscore. This result may lend support to the idea that some aspects of cognitive dysfunction are relevant contributors to migraine attack-related disability [16].

Taken together, our findings suggest that MwoA is associated with cognitive dysfunctions and in particular, that altered executive functioning can be related to high migraine related disability. Thus, these results underscore the importance of a careful evaluation of cognitive function even with a screening tool, provided that sensitive to detect dysfunctions of attention, memory, visuospatial and executive domains, as the MoCA is. Moreover, no correlation between scales assessing neuropsychiatric symptoms and cognitive tests suggests that the poorer cognitive performance exhibited by MwoA patients was not conditioned by the emotional variables such as depression, apathy or anxiety, differently from what reported in other neurological diseases [23, 40].

Our study had several strengths and limitations. The inclusion of a sample of patients homogeneous for type of migraine (all MwoA patients) and drug-naïve for preventive pharmacological therapies can be considered as strength of the study, suggesting that cognitive dysfunctions can occur in the natural history of MwoA, independently from preventive migraine therapies. As a consequence, these data could be considered important to better understand the MwoA clinical spectrum. However, the cross-sectional nature of our study did not allow us to ascertain whether the mild cognitive impairments we could detect here are amenable to changes related to pharmacological treatment, and can herald clinically relevant cognitive impairments. Our patient group was a convenience sample of treatment seeking migraine suffers and thus, may represent a limitation about generalizability of the findings. Furthermore, our sample was composed mainly of women as migraine is strongly related to gender, but this might also limit the generalizability of the results. Furthermore, we could not evaluate whether pain prevalence on either side of the head may be associated with different cognitive profiles, because most of our patients had no preferred side for pain. This issue deserved to be explored in further studies including balanced groups of patients with unilateral side of pain. Finally, in the present paper we only aimed at obtaining a screening of general cognitive functioning, and for this purpose we adopted a relatively recent tool, i.e. MoCA, which has been developed to obtain an estimation of several cognitive domains, including those (such as executive functions) that are often overlooked in more common screening tools. The MoCA cannot be used as a diagnostic instrument, but to screen cognitive status of patients attending a migraine ambulatory and to provide a baseline assessment, which could be compared to future assessments.

The cut-off score used in our study refers to age- and adjusted-scores and not to raw scores, and it is specifically foreseen by the available Italian normative study. The cut-off value is clearly lower than that reported in the original study based on a sample of 90 healthy Canadian controls (mean age 72.84 ± 7.03 years; mean education 13.33 ± 3.40), using a one-point correction for education (12 years). However, several factors likely contribute to this discrepancy such as: i) reference to age- and education-adjusted scores and not to raw scores; ii) use of population-based normative sample instead of convenience sample, as in the original study (note that even in English speaking countries population-based normative studies provided lower cut-off values than that proposed in the original study [41]; iii) cultural and linguistic biases related to translations of the test in different languages (as in Portuguese [42] and Japan language [43]. For this reasons it has been clearly stated that appropriate MoCA normative data have to be employed when interpreting MoCA scores [41]. It is also interesting to note that the cut-off value of 15.5 for age- and education-adjusted scores did not identify any control or migraineurs participant as affected by cognitive impairment, whereas applying the cut-off score of 26 cognitive impairment would be present in 68/72 (94.4 %) MwoA patients and in 44/72 (61.1 %) controls. Such percentages appear to be implausibly high in two samples of home-dwelling and active young adults, thus further reinforcing the need for cut-off points based upon country-specific normative data.

## Conclusions

In conclusion, we believe that early identification of cognitive deficits in MwoA patients is relevant for future care planning. The MoCA seems suitable to screen such cognitive defects in clinical practice in MwoA patients.

## Abbreviations

AES-S: Self-version of apathy evaluation scale
BDI-II: Beck depression inventory-II
HCs: healthy controls
HIT-6: Headache impact test −6
MIDAS: Migraine disability assessment scale
MoCA: Montreal cognitive assessment
MwA: Migraine with aura
MwoA: Migraine without aura
SPSS: Statistical package for the social sciences
STAI-Y-1: State anxiety inventory
STAI-Y-2: Trait anxiety inventory
VAS: Visual analogic scale
